# Rare case of vascular ring with Kommerell diverticulum mimicking uncontrolled asthma

**DOI:** 10.1002/rcr2.535

**Published:** 2020-02-19

**Authors:** Ahmed Ben Saad, Nesrine Fahem, Houda Ben Saad, Asma Migaou, Chokri Kortas, Samah Joobeur

**Affiliations:** ^1^ Pulmonology Department Fattouma Bourguiba Teaching Hospital Monastir Tunisia; ^2^ Medical Department, Cardiology Division Moknine Hospital Monastir Tunisia; ^3^ Department of Cardiovascular and Thoracic Surgery Sahloul University Hospital Sousse Tunisia

**Keywords:** Aortic diseases, asthma, diverticulum, surgery, vascular ring

## Abstract

Anomalies of the aortic arch associated with Kommerell diverticulum (KD) are rare congenital malformations. Symptomatic thoracic vascular rings presenting in adults are rare. We report a case of a 39‐year‐old woman who was diagnosed with uncontrolled asthma. She was complaining of worsening respiratory symptoms with dysphagia. Imaging studies and preoperative findings concluded to type II congenital anomaly of the aortic arch or Neuhauser's anomaly: a right‐sided aortic arch with aberrant left subclavian artery, tracheoesophageal compression by KD and ligamentum arteriosum (LA). This compression was relieved by the resection of the LA and KD.

## Graphical abstract

We report a rare case of vascular ring with Kommerell diverticulum leading to a misdiagnosis of asthma in a 39‐year‐old woman.

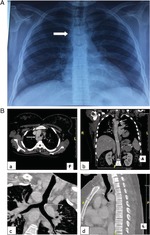

## Introduction

Right‐sided aortic arch associated with a Kommerell diverticulum (KD), an aberrant left subclavian artery and a ligamentum arteriosum (LA) is one of the congenital anomalies of the aortic arch or vascular rings. It is a rare condition estimated to occur in 0.1% of the general population 1. This morphological abnormality is also called Neuhauser vascular anomaly. It consists of an ascending aorta in normal position, with a right‐sided aortic arch running to the right of the trachea and oesophagus. The LA extends from the left pulmonary artery to the aorta at the isthmus leading to a compression of the oesophagus and trachea with the appearance of an aneurysmal diverticulum of the descending aorta called KD. The dilatation of the latter results in compression of the surrounding structures 1, 2, 3. Symptomatic thoracic vascular rings presenting in adults are rare. Furthermore, clinical manifestations are uncommon. It may lead to a tracheoesophageal compression with different symptoms like dyspnoea, dysphagia, stridor, wheezing, or recurrent respiratory infections 2. Tracheal compression may simulate asthma and may cause the delay of the diagnosis 1.

## Case Report

A 39‐year‐old woman, with a history of non‐steroidal anti‐inflammatory induced hypersensitivity, was referred from primary care to our department to investigate a suspected “uncontrolled asthma.” She was working in a garment factory for 17 years and was a non‐smoker. She reported chest tightness, dyspnoea on exertion with progressive worsening in the last year, and chronic dry cough for four years. She was also complaining of dysphagia that started one year ago. She had been under inhaled treatments (corticosteroids, short and long acting bronchodilatators) for approximately three years without symptoms relieve.

**Figure 1:**
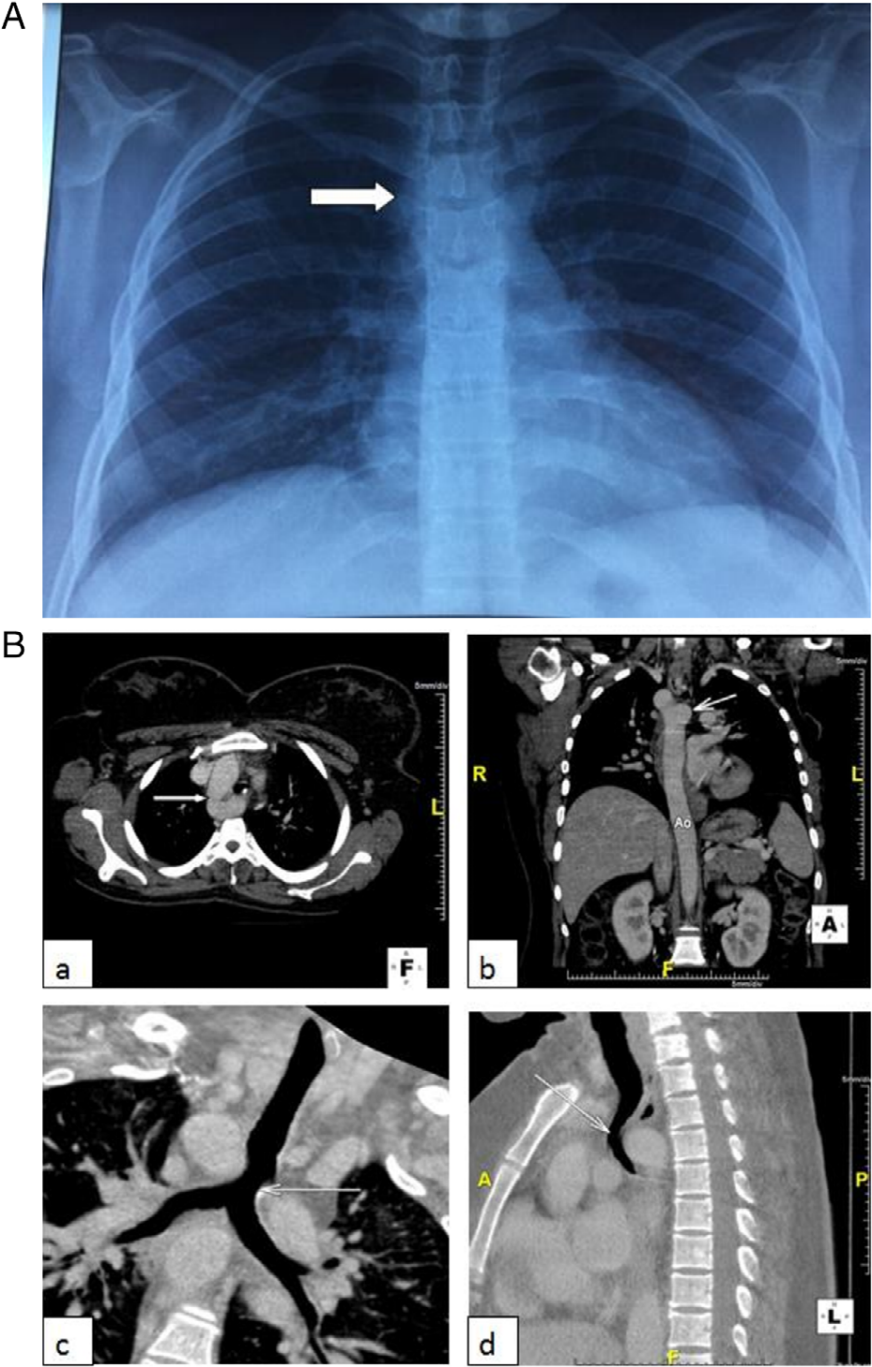
Imaging findings: (A) Frontal chest radiograph: right‐sided aortic arch with a right‐sided aortic knob (white arrow). (B) Chest CT Scan: a right‐sided aortic arch and a tracheal compression due to the diverticulum of Kommerell. (a) Axial image: right‐sided aortic arch (white arrow). (b) Coronal image: diverticulum of Kommerell (white arrow). (c) Coronal image (reconstruction): tracheal compression (white arrow). (d) Sagittal image: tracheal compression (white arrow).

The physical examination was normal. Blood tests (compete blood count, chemistry, total IgE) were within normal limits. Pulmonary function tests were normal. On the chest radiograph, the aortic knob was located on the right side of the trachea (Fig. 1A). Fiberoptic bronchoscopy was not tolerated by the patient. The thoracic computed tomography (CT) scan revealed the presence of a right‐sided aortic arch, a common origin of the left subclavian artery and the left common carotid, a diverticular dilatation of the initial portion of the descending aorta corresponding to the diverticulum of Kommerell (KD), and a squeeze on the trachea and the oesophagus (Fig. 1B). Echocardiography excluded other associated cardiac malformations. The diagnosis of type II congenital anomaly of the aortic arch was retained.

**Figure 2:**
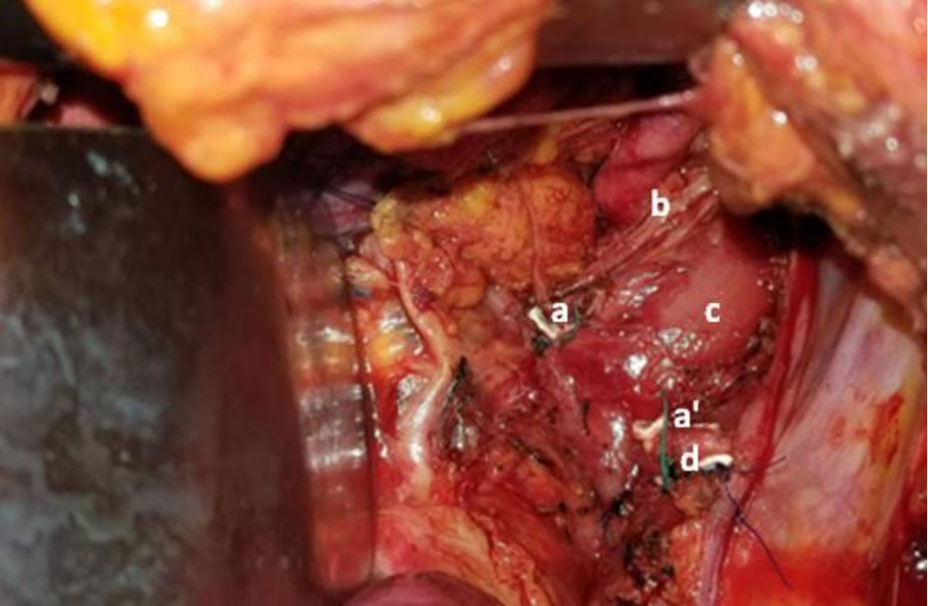
Peroperative image: resection of the ligamentum arteriosum. (a and a′) Extremities of the ligamentum arteriosum after resection, (b) vagus nerve, (c) oesophagus, (d) descending aorta.

Given the worsening of the symptoms and the risk of rupture of the KD, surgical treatment was indicated. The patient underwent posterolateral left thoracotomy. Preoperative findings showed an aberrant left subclavian artery, a pseudoaneurysmal dilatation of the descending thoracic aorta corresponding to the KD, and a posterior squeeze of the oesophagus and the trachea by the LA (ductus arteriosus). A resection of the LA and the KD was performed (Fig. 2). Following surgery, the patient presented dysphonia due to a left laryngeal nerve paralysis improved by orthophonic physiotherapy. She has no more reported dysphagia or respiratory symptoms.

## Discussion

Right‐sided aortic arch with aberrant left subclavian artery and KD represents type II congenital anomaly of the aortic arch or Neuhauser's anomaly 2. Aortic arch anomalies are secondary to abnormal embryologic development occurring between the third and eighth weeks of gestation, during the development of the thoracic aorta and the main arterial trunks 2. There are different anatomic variations. The KD is an embryologic remnant of the left fourth aortic arch 2.

This congenital abnormality is characterized by a wide disease spectrum, ranging from prenatally detected cases to asymptomatic clinical course or serious complications 2, 3. In fact, some cases are discovered incidentally. The KD and the LA may cause compression of the trachea or the oesophagus causing either respiratory symptoms such dyspnoea, chest pain, stridor, and low respiratory tract infections, or dysphagia 1. It might take years before associating symptoms to the aortic arch anomaly.

Respiratory symptoms as asthmatic dyspnoea or misdiagnosis of asthma are less frequent. Only few cases of patients diagnosed initially as asthma before making the diagnosis of congenital anomaly of the aortic arch are reported in the literature 1. In fact, our patient was unsuccessfully treated as asthma during four years. Thus, performing a chest X‐ray to newly diagnosed asthmatic patients is required.

Investigations that traditionally have been used for the diagnosis of these anomalies are barium oesophagography, angiography, and echocardiography. Angiography is an invasive procedure and it is replaced nowadays by non‐invasive diagnostic tools like magnetic resonance imaging (MRI) and CT 2. The barium oesophagography shows the oesophageal compression due to the vascular anomaly 4. Echocardiography is performed to rule out associated cardiac pathology 2. Bronchoscopy can show an extrinsic tracheal compression. It permits also to eliminate intrinsic disease of the airway. Thoracic CT scan or MRI is accurate and non‐invasive examinations that should be indicated to diagnose vascular abnormalities 2. In our case, thoracic CT scan confirmed the diagnosis of this congenital anomaly. The latest technique added to the diagnostic tools is foetal echocardiography. It has facilitated the diagnosis of aortic arch anomalies in the foetal stage 3.

Surgical indications for a right aortic arch, an aberrant left subclavian artery, a residual LA associated to a KD have not been well defined because of the rareness of the condition. Main indications are a symptomatic condition, the size of the diverticulum, and a high risk of complications. Left posterolateral thoracotomy represents the better approach. The resection of the LA may be enough to decompress the trachea and the oesophagus 1. The resection of the KD is discussed. Some authors consider that the indication of resection is related to the size of the diverticulum, since the resection of the LA may suffice to make it disappear. However, other authors recommend a systematic resection due to the risk of an aneurysmal transformation rupture 5. In fact, a diverticulum may become aneurysmal even after the LA resection 5. Furthermore, the resection of the LA alone may not be sufficient to relieve compression symptoms 1. In some cases, the resection and reimplantation of the left subclavian artery is performed.

The postsurgical outcomes are usually favourable. Some complications are described such tracheomalacia secondary to a chronic compression of the trachea or atelectasis. Left recurrent laryngeal nerve paralysis has rarely been described 1. Mortality rates related to this surgery are ranging from zero to 5% according to some reported series 1. Symptomatic improvement is obtained in more than 90% of operated patients 5.

In conclusion, although rare, congenital anomalies of the aortic arch with KD should be considered in the differential diagnosis of uncontrolled asthma in particular if associated to oesophageal symptoms. Chest CT scan is a non‐invasive procedure to confirm the diagnosis. Surgical treatment is a safe and efficient way to relieve symptoms and avoid complications.

### Disclosure Statement

Appropriate written informed consent was obtained for publication of this case report and accompanying images.
